# Gender Differences between Multimorbidity and All-Cause Mortality among Older Adults

**DOI:** 10.1155/2020/7816785

**Published:** 2020-02-19

**Authors:** Alejandra Andrea Roman Lay, Carla Ferreira do Nascimento, Fresia Caba Burgos, Angélica del Carmen Larraín Huerta, René Eduardo Rivera Zeballos, Verónica Pantoja Silva, Yeda Aparecida de Oliveira Duarte

**Affiliations:** ^1^Faculty of Health Sciences, University of Tarapacá, Arica, Chile; ^2^Department of Epidemiology, School of Public Health, University of São Paulo, São Paulo, Brazil; ^3^Facultad de Salud, Universidad Bernardo O'Higgins, Santiago, Chile; ^4^Universidad de Santiago de Chile, Santiago, Chile; ^5^Hospital Félix Bulnes Cerda, Universidad de Santiago de Chile, Santiago, Chile; ^6^Núcleo Ciencias Biológicas, Facultad de Estudios Interdisciplinarios, Universidad Mayor, Santiago, Chile; ^7^School of Nursing, University of São Paulo, São Paulo, Brazil

## Abstract

**Objectives:**

This study seeks to determine the prevalence of chronic diseases and analyze the association between multimorbidity and all-cause mortality by sex.

**Methods:**

This is a 16-year longitudinal study of follow-up. We used sample data of the SABE (Health, Well-Being and Aging) study cohort and mortality data obtained through the Mortality Information Improvement Program of the City of São Paulo (PRO-AIM) from the 2000–2016 period. Survival analysis was performed using Cox proportional hazard models.

**Results:**

Hypertension (HT) was the most prevalent disease in older adults (52.93%), followed by musculoskeletal disorders (MSDs) (27.09%), cardiovascular diseases (CD) (17.79%), diabetes mellitus (DM) (16.95%), mental disorders (MD) (15.43%), and respiratory diseases (RD) (9.72%). The highest mortality rate in women was observed in the combination of HT/MSDs/DM/MD (HR = 6.15, 95% CI = 2.32, 16.32), while in men was in the combination of HT/CD/MSDs/DM (HR = 5.72, 95% CI = 1.72, 19.06).

**Conclusion:**

Similar to previous studies carried out in developed countries, we found that all-cause mortality increased as diseases are added to an individual. Women and men presented different mortality patterns according to multimorbidity. Therefore, we suggest that additional longitudinal studies should be performed in order to analyze mortality by sex.

## 1. Introduction

Given the worldwide decrease in fertility rates and increase in life expectancy, aging population is growing in almost all countries [1]. Global average life expectancy increased 5.5 years from 2000 to 2016 [2, 3]. One of the main consequences of aging is the increase in elderly population with chronic Multimorbidity (MM), which is greater in low- and middle-income countries (LMICs) [4]. As defined by the World Health Organization (WHO), MM is when two or more chronic diseases coexist at the same time in an individual [5]. The European General Practitioners Research Network (EGPRN) mentions that the addition of another disease can be acute or chronic. The inclusion of a biopsychosocial factor, associated or not with the previous disease or with a somatic risk factor [6], can lead to a great heterogeneity of MM definitions among studies, mainly in terms of risk factors [7]. MM has been associated with adverse health outcomes such as poorer quality of life [8], low physical function [9], and greater demand of healthcare cost [10, 11]. Additionally, MM has been associated with an increased risk of all-cause mortality. However, results have been analyzed with caution since excess risk may depend on disease combinations [12–14] with or without associated risk factors [15].

There are no studies analyzing MM and mortality in Latin America. Therefore, we decided to evaluate MM in a representative sample using data from the SABE study. The goals of this study are as follows: (1) to determine the prevalence of individual chronic diseases and of the five most prevalent combinations of two, three, four, and five chronic diseases and (2) to analyze the association between multimorbidity in the most prevalent combinations and all-cause mortality by sex.

## 2. Methods

### 2.1. Sample

The SABE methodology has been described elsewhere [16, 17]. It was a cross-sectional study organized by the Pan American Health Organization (PAHO/WHO) in 2000 and involved the participation of seven Latin American countries. Its objective was to determine the living conditions of elderly people aged 60 or more. In 2000, the study became a cohort study in the São Paulo municipality [16]. Nowadays, it has four waves of follow-up (2000, 2006, 2010, and 2015). In this study, we used the first wave sample which was taken in two segments. The first segment was a probabilistic sample that used 72 census tracts from 263 sectors. These were drawn by the conglomerate sample from the National Household Sample Survey (PNAD), and the sample selected 1,568 older adults. The second segment selected 575 residents of the municipalities where the previous interviews were conducted. Thus, 2,143 participants were interviewed at the end of the first wave [18]. The sample size weights were adjusted according to the characteristics of the population in terms of age and sex, and the size of the population aged 60 years or more was registered by the Brazilian Institute of Geography and Statistics [19].

### 2.2. Data Collection

The original survey questionnaires created by the PAHO were translated into Portuguese for their use in Brazil. The survey was conducted by trained interviewers. The SABE study was approved by the Research Ethics Committee of the Faculty of Public Health of the University of São Paulo (FSP-USP) and the National Research Ethics Commission (CONEP).

### 2.3. Outcomes

Our first outcome was the prevalence of individual chronic diseases and multimorbidity, which is defined as the presence of two or more chronic diseases in an individual. Our second outcome was all-cause mortality according to multimorbidity. Mortality data were obtained through linkage, which was performed between the database of the Program for Improvement of Mortality Information in the municipality of São Paulo (PRO-AIM) (cutoff date: September 13, 2016) and the 2000 cohort of SABE. We obtained the underlying cause of death through death certificates, and these were categorized according to the International Classification of Diseases (ICD), 10^th^ revision.

### 2.4. Independent Variables

The independent variables were obtained through the SABE questionnaire used in 2000. We used socioeconomic variables such age (years), education (without, 1 to 7, 8, or more years), marital status (married, single, widow, separated/divorced), and living arrangement (living with someone, living alone). Health-related variables such as self-rated health (excellent/very good/good, fair/poor), visual impairment (excellent/very good/good, fair/poor/blind), and mini-mental score (13 or more, 12 or less). The chronic diseases were self-reported and categorized as yes or no; Hypertension (HT), Diabetes Mellitus (DM), Musculoskeletal Disorders (MSDs) (osteoarthritis, rheumatoid arthritis), Cardiovascular Diseases (CD) (coronary heart disease, congestive heart failure, angina), Mental Disorders (MD) (psychiatric disorder), and Respiratory Diseases (RD) (chronic obstructive pulmonary disease, asthma).

### 2.5. Data Analysis

In 2000, of the 2,143 participants interviewed, 878 were men and 1,265 were women. In 16 years of follow-up, a total of 1,374 deaths were obtained. We excluded 40 deaths due to external causes and 99 deaths missing from the variables of interest such as education (*n* = 12), marital status (*n* = 1), self-rated health (*n* = 4), visual impairment (*n* = 9), hypertension (*n* = 14), musculoskeletal disorders (*n* = 29), cardiovascular (*n* = 11), diabetes mellitus (*n* = 10), mental disorders (*n* = 4), and respiratory diseases (*n* = 5), which led to 2,004 participants for the analysis. For data without death information, it was censored until the last observation in September 13, 2016. Hence, of the total number of individuals, 1,263 were deaths and 741 individuals were censored.

In order to obtain the prevalence of diseases, we first calculated the prevalence of each chronic disease and then for all possible combinations of two chronic diseases. The five most prevalent pairs were used to form the combination of three chronic diseases. Then, the first five most prevalent trios were used to form the combination of four diseases and so on until we reached five combinations. All-cause mortality was calculated according to combinations of two, three, four, and five prevalent diseases.

We used the sample weight of the 2000 wave throughout the analysis, and we performed the Rao–Scott chi-square test. The proportional hazard assumption was tested by Schoenfeld residuals. The all-cause mortality results were adjusted by age, education, marital status, living arrangement, self-rated health, visual impairment, and mini-mental score, and then, these were stratified by sex.

## 3. Results

In Table 1, the mean ages of women and men were 72.76 and 73.50, respectively. There were statistically significant differences between women and men according to education (*p* < 0.001), marital status (*p* < 0.001), living arrangement (*p* < 0.001), and visual impairment (*p* < 0.05). Regarding chronic diseases, women had higher prevalence of diseases when compared to men with the exception of CD and RD; HT (55.79% versus 48.82%), MSDs (34.89% versus 15.84%), DM (17.53% versus 16.12%), MD (17.40% versus 12.58%), CD (17.14% versus 18.73%), and RD (8.30% versus 11.76%).

### 3.1. Prevalence of Multimorbidity

The five most prevalent pairs were HT/MSDs (16.25%), HT/CD (13.06%), HT/DM (12.78%), HT/MD (8.14%), and CD/MSDs (6.67%) (Table 2). With regard to the five most prevalent trios, the highest prevalence corresponded to HT/CD/MSDs (4.68%), while the lowest one to HT/CD/MD (2.19%). When it came to quartets, the most prevalent was HT/CD/MSDs/DM (1.03%), while the least one was HT/CD/DM/MD (0.51%). For quintets, the most prevalent was HT/CD/MSDs/DM/MD (0.20%), while the least one was HT/CD/MSDs/DM/RD and HT/MSDs/DM/MD/RD (0.13% each). There were no values for the HT/CD/DM/MD/RD combination (Table 3).

### 3.2. All-Cause Mortality and MM

#### 3.2.1. Combination of Two Chronic Diseases

Women with two diseases had an increased risk of all-cause mortality for all combinations, with a wide range from 36% to 105% higher risk in relation to women without diseases (HT/MSDs: HR = 1.36, 95% CI = 1.01, 1.82, CD/MSDs: HR = 1.48, 95% CI = 1.10, 1.98, HT/MD: HR = 1.53, 95% CI = 1.09, 2.13, HT/DM: HR = 1.82, 95% CI = 1.36, 2.44, and HT/CD: HR = 2.05, 95% CI = 1.52, 2.78). However, men had 58% to 59% increased risk of all-cause mortality when compared to men without diseases (HT/DM: HR = 1.58, 95% CI = 1.09, 2.30, and CD/MSDs: HR = 1.59, 95% CI = 1.06, 2.38, respectively) (see Table 4).

#### 3.2.2. Combination of Three Chronic Diseases

Women with three diseases had risk of all-cause mortality twice as high as women without diseases (HT/CD/MD: HR = 2.11, 95% CI = 1.20, 3.72, HT/CD/MSDs: HR = 2.26, 95% CI = 1.55, 3.30, and HT/CD/DM: HR = 2.46, 95% CI = 1.53, 3.95). Similarly, men doubled the risk in HT/MSDs/DM and HT/CD/DM combinations (HR = 2.36, 95% CI = 1.24, 4.48 and HR = 2.53, 95% CI = 1.37, 4.66, respectively) (Table 4).

#### 3.2.3. Combinations of Four Diseases

In Table 5, women with four diseases had risk of all-cause mortality twice as high as women without diseases (HT/CD/MSDs/MD: HR = 2.16, 95% CI = 1.04, 4.51, HT/MSDs/DM/MD: HR = 2.27, 95% CI = 1.18, 4.38, and HT/CD/MSDs/RD: HR = 2.97, 95% CI = 1.68, 5.25). The exception to this was the HT/CD/DM/MD combination, where women had four times the risk of all-cause mortality (HR: 4, 56, 95% CI: 1.69, 12.29). However, men had 162% to 472% higher risk of all-cause mortality in relation to men without diseases (HT/MSDs/DM/MD: HR = 2.62, 95% CI = 1.85, 3.70, HT/CD/MSDs/RD: HR = 2.86, 95% CI = 1.18, 6.88, HT/CD/MSDs/MD: HR = 3.81, 95% CI = 2.04, 7.08, and HT/CD/MSDs/DM: HR = 5.72, 95% CI = 1.72, 19.06).

#### 3.2.4. Combinations of Five Diseases

Women presented two combinations of five diseases with higher risk of all-cause mortality when compared to women without diseases (HT/CD/MSDs/MD/RD: HR = 4.14, 95% CI = 1.24, 13.85 and HT/CD/MSDs/DM/MD: HR = 6.15, 95% CI = 2.32, 16.32). However, men only showed one combination with increased risk of all-cause mortality (HT/CD/MSDs/DM/MD: HR = 2.94, 95% CI = 2.02, 4.29). There were no observations in other combinations (Table 5).

## 4. Discussion

The female sex is associated with increased risk of MM [20], which is similar to our results where women presented higher prevalence of each disease individually. There was a higher percentage of women without education than men without education (21.40% versus 15.83%). In a previous meta-analysis, lower education was related to MM [21]. This is concerning since Brazil continues to be one of the countries with greater inequalities worldwide [22], despite the improvements that have been made over the last years.

Similar to previous studies, our results show that hypertension was the most prevalent disease in older adults [23, 24]. It is the greater risk factor for cardiovascular disease and increases the risk of cardiovascular mortality. In high-income countries, the prevalence of hypertension is decreasing by 2.6%; however, in low- and middle-income countries, this prevalence is increasing by 7.7% [25]. In this study, in a representative sample of the Brazilian population, both women and men presented hypertension across most of the prevalent disease combinations, with the exception of the combination of musculoskeletal disorders and cardiovascular disease.

Our results are similar to the ones obtained for older adults from Northeast China, where the pair combination of hypertension and cardiovascular diseases was the second most prevalent among older adults [26]. In women, the most prevalent individual diseases were (from highest to lowest) hypertension, musculoskeletal disorders, and diabetes. Hypertension was present in all combinations with the highest mortality. Women had risk of all-cause mortality twice as high when they had hypertension and cardiovascular diseases, and this risk increased even more with the addition of other diseases to this pair. After the addition of diabetes mellitus, this risk remained twice as high. However, it quadrupled with mental disorders, and the risk of all-cause mortality had a six-fold increase when musculoskeletal disorders were added. A possible mechanism that explains the increased risk in women is that participants in the SABE study were of 60 years or more when they were interviewed, which means that they were all postmenopausal at the beginning of the study. Postmenopausal women have higher risk of hypertension and diabetes mellitus, which increases the risk for cardiovascular diseases [27]. This occurs because estrogen acts as a protective factor in premenopausal women, but this protection disappears after menopause [28]. Furthermore, women with hypertension had increased risk for cardiovascular mortality. Previous studies show that women with hypertension had increased risk for coronary heart disease (CHD) and stroke mortality [29]. Additionally, women with hypertension and ischemic heart disease (IHD) had increased risk for cardiovascular mortality [30].

There was an increased risk of all-cause mortality when mental disorder was added. Women had more prevalence of self-reported MD when compared to men, a similar result to those obtained by other studies showing that women had higher prevalence of mental illness such as depression [31] and anxiety [32]. Furthermore, the presence of comorbidity of hypertension and cardiovascular diseases increases the incidence of depression [33]. Additionally, women from the SABE study presented higher prevalence of factors related to MD including marital status of widow and self-report of living alone. Thus, women from our sample could be more likely to suffer from mental disorders such as depression. It is known that mental disorders have been associated with increased risk of mortality. One study in Finland demonstrated that women with psychotic disorders had higher risk of all-cause mortality in relation to men [34], while other study shows that depression increased the risk of cardiovascular mortality [33]. Furthermore, another study found that people with diabetes and depression had higher risk of all-cause mortality when compared to people suffering only from diabetes [35]. Moreover, by itself, mental illness increased the risk of premature mortality, which can be accelerated by cardiovascular diseases [36]. Finally, with the addition of some MSDs, the risk of all-cause mortality has a six-fold increase. In a similar fashion to mental disorder, some MSDs such as knee osteoarthritis (OA) or generalized OA increased the risk of all-cause mortality [37] by themselves. Therefore, when all diseases interacted together in a single individual, they gradually increased the risk of all-cause mortality.

The individual disease with the highest prevalence in men was hypertension, followed by cardiovascular diseases and diabetes mellitus. However, the highest mortality was found in the combination of cardiovascular diseases and musculoskeletal disorders, which formed pair, quadruple, and quintuple diseases. The exception to this was trios, where the highest mortality, as in women, was the hypertension, cardiovascular diseases, and diabetes mellitus combination. There was no risk of all-cause mortality due to respiratory diseases. Men with CD and MSDs had 59% higher risk of all-cause mortality in relation to men without diseases. One study with 14 years of follow-up demonstrated that elderly people with cardiovascular disease and arthritis had a decreased median survival (8.2 years) in relation to people without chronic diseases (10.3 years) [12]. According to our results, although the prevalence of MSDs was higher in women than in men, the increased all-cause mortality due to MSDs was higher in men than in women. These results may be explained by the fact that the men in our sample shared factors related to osteoarthritis and increased risk of all-cause mortality such as alcohol consumption and sedentary behavior, besides older age. Of the people who do not drink, 32% were males. Of the people who reported drinking every day, 83% were males. Regarding sedentary behavior, 70% of men reported not doing any physical activity. Although most men reported being sedentary, they did not have a BMI of overweight or obesity, a factor highly correlated to OA of the knee and hip; 43% of men had a normal body mass index (BMI) (data not shown). Chronic alcohol consumption could increase inflammatory processes affecting the knee and shoulder joints, generating OA or worsening the existing inflammation [38]. While sedentary behavior negatively affects musculoskeletal conditions such as knee OA [39], it is known that physical activity produces benefits to the cartilage, bone, muscles, and tendons in men [40]. We could not establish if the sedentary behavior was due to either a painful OA that limited physical exercise or the burden of existing chronic diseases in the individual. Additionally, men in our sample had a high prevalence of chronic respiratory diseases. This could generate dyspnea, limit the performance of daily life activities, and therefore, increase a sedentary lifestyle. Similarly, we could not establish whether individuals were first affected by musculoskeletal or cardiovascular disease. The latter also generates dyspnea, which could increase the respiratory difficulty of a basic chronic disease, thereby increasing a sedentary behavior. What we can establish is that sedentary behavior increases the risk of all-cause mortality. A recent meta-analysis found that sedentary behavior above eight hours per day had a linear tendency associated with an increased risk of all-cause mortality [41].

This study has limitations. First, we could not establish which disease started first in an individual. This is a retrospective study, and for each disease, we did not know the onset (to establish which one started first) and the severity. Furthermore, we did not have information about the diagnostic of mental disorders.

Second, given that the inclusion of diseases and risk factor changes between studies and countries, we could compare our results with few studies. Furthermore, there was one study with multimorbidity variable as number of diseases, that is, 1, 2, 3, etc. [42], and other studies that do not separate diseases by type include multimorbidity as a continuous variable in the survival model [42–44], making it complex to compare results between studies. Finally, there were no studies that analyzed mortality results by sex.

Despite these limitations, multimorbidity was obtained in a community sample that is representative of the Brazilian elderly population. We believe this is the first cohort study in Latin America evaluating multimorbidity with all-cause mortality and the first study analyzing this association by sex.

## 5. Conclusion

We have shown that mortality in older adults increased with the addition of diseases. This is of utmost importance since currently the clinical guidelines are focused on the management of diseases individually and not on the sum of them. We also observed that men and women should be analyzed separately, given that women and men presented differences in risk factors and prevalence of diseases, which was reflected in the mortality.

## Figures and Tables

**Table 1:** Baseline characteristics by sex (SABE study, municipality of São Paulo, 2000).

	Female	Male	All	*p* value^a^
Age, mean (SD)	72.76 (8.43)	73.50 (8.53)	73.06 (8.48)	
Education: without	21.40	15.83	19.12	<0.001
Marital status: widow	42.07	10.25	29.02	<0.001
Living arrangement: living alone	16.80	7.61	13.04	<0.001
Self-rated health: fair/poor	55.27	51.34	53.66	0.09
Visual impairment: fair/poor/blind	61.49	53.07	58.04	0.003
Mini mental score: 12 or less	12.93	12.06	12.57	0.59
Hypertension	55.79	48.82	52.93	0.009
Musculoskeletal disorders	34.89	15.84	27.09	<0.001
Cardiovascular diseases	17.14	18.73	17.79	0.39
Diabetes mellitus	17.53	16.12	16.95	0.46
Mental disorders	17.40	12.58	15.43	0.01
Respiratory diseases	8.30	11.76	9.72	0.02

^*a*^ *p* value of Rao–Scott chi-square test.

**Table 2:** Prevalence of the combination of two chronic diseases (SABE study, municipality of São Paulo, 2000).

	HT	MSDs	CD	DM	MD	RD
HT	†	—	—	—	—	—
MSDs	^*∗*^16.25	†	—	—	—	—
CD	^*∗*^13.06	^*∗*^6.67	†	—	—	—
DM	^*∗*^12.78	5.03	3.94	†	—	—
MD	^*∗*^8.14	5.10	3.15	2.81	†	—
RD	5.00	3.02	2.73	1.23	1.66	†

HT: hypertension; MSDS: musculoskeletal disorders; CD: cardiovascular diseases; DM: diabetes mellitus; MD: mental disorders; RD: respiratory diseases. ^*∗*^The most prevalent five combinations. † denotes no value. — denotes values which were previously calculated.

**Table 3:** Highest prevalence^*∗*^ of chronic diseases (SABE study, municipality of São Paulo, 2000).

3 chronic diseases	Prevalence	4 chronic diseases	Prevalence	5 chronic diseases	Prevalence
HT-CD-MSDs	^*∗*^4.68	HT-CD-MSDs-DM	^*∗*^1.03	HT-CD-MSDs-DM-MD	^*∗*^0.20
HT-MSDs-DM	^*∗*^3.95	HT-CD-MSDs-MD	^*∗*^0.94	HT-CD-MSDs-DM-RD	^*∗*^0.13
HT-MSDs-MD	^*∗*^2.76	HT-CD-MSDs-RD	^*∗*^0.62	HT-CD-MSDs-MD-RD	^*∗*^0.15
HT-CD-DM	^*∗*^3.12	HT-MSDs-DM-MD	^*∗*^0.92	HT-MSDs-DM-MD-RD	^*∗*^0.13
HT-CD-MD	^*∗*^2.19	HT-CD-DM-MD	^*∗*^0.51	HT-CD-DM-MD-RD	†

^*∗*^The most five prevalent combinations of chronic diseases. HT: hypertension; MSDS: musculoskeletal disorders; CD: cardiovascular diseases; DM: diabetes mellitus; MD: mental disorders; RD: respiratory diseases. † denotes no value.

**Table 4:** Adjusted hazard ratios^*∗*^ (95% confidence intervals) for all-cause mortality according to combinations of two and three chronic diseases by sex (SABE study, municipality of São Paulo, 2000).

Combinations of diseases	By sex			
	Females		Males	
*Two diseases* ^*a*^	HR	(95% CI)	HR	(95% CI)
HT-MSDs	1.36	1.01–1.82	1.15	0.77, 1.71
One of two	1.42	1.16–1.74	1.21	0.98–1.49
HT-CD	2.05	1.52, 2.78	1.44	0.98, 2.10
One of two	1.30	1.07, 1.59	1.06	0.83, 1.35
HT-DM	1.82	1.36, 2.44	1.58	1.09, 2.30
One of two	1.40	1.14, 1.70	1.07	0.86, 1.36
HT-MD	1.53	1.09, 2.13	1.04	0.82, 1.32
One of two	1.41	1.17, 1.70	0.85	0.53, 1.37
CD-MSDs	1.48	1.10, 1.98	1.59	1.06, 2.38
One of two	1.09	0.90, 1.34	1.43	1.09, 1.89

*Three diseases* ^*a*^				
HT-CD-MSDs	2.26	1.55, 3.30	1.49	0.83, 2.67
Two of three	1.41	1.06, 1.87	1.41	1.00, 2.01
One of three	1.48	1.19, 1.83	1.19	0.96, 1.47
HT-MSDs-DM	1.48	0.99, 2.20	2.36	1.24, 4.48
Two of three	1.70	1.30, 2.20	1.41	1.01, 1.98
One of three	1.34	1.08, 1.67	1.18	0.96, 1.46
HT-MSDs-MD	1.42	0.84, 2.39	1.88	0.74, 4.79
Two of three	1.47	1.13, 1.91	1.03	0.71, 1.50
One of three	1.49	1.21, 1.82	1.19	0.94, 1.51
HT-CD-DM	2.46	1.53, 3.95	2.53	1.37, 4.66
Two of three	1.91	1.45, 2.52	1.39	1.02, 1.89
One of three	1.27	1.06, 1.53	1.18	0.93, 1.50
HT-CD-MD	2.11	1.20, 3.72	1.72	0.78, 3.76
Two of three	1.83	1.39, 2.41	1.13	0.81, 1.59
One of three	1.37	1.15, 1.64	1.07	0.83, 1.38

^*∗*^Multivariate Cox proportional hazards models adjusted by age, education, marital status, living arrangement, self-rated health, visual impairment, and mini-mental score. ^a^Referent: no disease. HT: hypertension; MSDS: musculoskeletal disorders; CD: cardiovascular diseases; DM: diabetes mellitus; MD: mental disorders; RD: respiratory diseases.

**Table 5:** Adjusted hazard ratios^*∗*^ (95% Confidence intervals) for all-cause mortality according to combinations of four and five chronic diseases by sex (SABE study, municipality of São Paulo, 2000).

Combinations of diseases	By sex			
	Females		Males	
*Four diseases* ^*a*^	HR	(95% CI)	HR	(95% CI)
HT-CD-MSDs-DM	1.74	0.95, 3.19	5.72	1.72, 19.06
Three of four	2.40	1.66, 3.48	1.83	1.21, 2.73
Two of four	1.50	1.15, 1.95	1.45	1.06, 2.00
One of four	1.46	1.17, 1.81	1.24	0.98, 1.55
HT-CD-MSDs-MD	2.16	1.04, 4.51	3.81	2.04, 7.08
Three of four	1.91	1.32, 2.76	1.38	0.77, 2.48
Two of four	1.59	1.21, 2.09	1.22	0.89, 1.69
One of four	1.53	1.23, 1.89	1.19	0.93, 1.52
HT-CD- MSDs-RD	2.97	1.68, 5.25	2.86	1.18, 6.88
Three of four	2.16	1.50, 3.10	1.80	1.11, 2.91
Two of four	1.44	1.07, 1.92	1.50	1.08, 2.08
One of four	1.46	1.16, 1.82	1.22	0.97, 1.54
HT-MSDs-DM-MD	2.27	1.18, 4.38	2.62	1.85, 3.70
Three of four	1.43	1.01, 2.01	1.56	0.83, 2.95
Two of four	1.78	1.39, 2.29	1.34	0.96, 1.85
One of four	1.42	1.15, 1.75	1.20	0.95, 1.51
HT-CD-DM-MD	4.56	1.69, 12.29	1.84	0.44, 7.63
Three of four	2.14	1.51, 3.04	1.89	1.06, 3.40
Two of four	1.84	1.39, 2.44	1.29	0.95, 1.74
One of four	1.38	1.14, 1.67	1.24	0.97, 1.59

*Five diseases* ^*a*^				
HT-CD-MSDs-DM-MD	6.15	2.32, 16.32	2.94	2.02, 4.29
Four of five	1.99	1.27, 3.12	3.63	0.94, 13.96
Three of five	2.36	1.66, 3.36	1.47	0.94, 2.32
Two of five	1.71	1.32, 2.21	1.35	0.99, 1.83
One of five	1.52	1.22, 1.91	1.28	0.99, 1.63
HT-CD-MSDs-DM-RD	3.65	0.91, 14.57	†	
Four of five	1.97	1.25, 3.10	4.60	2.62, 8.09
Three of five	2.57	1.76, 3.75	1.55	0.94, 2.53
Two of five	1.51	1.13, 2.00	1.61	1.17, 2.20
One of five	1.41	1.11, 1.79	1.27	0.97, 1.66
HT-CD-MSDs-MD-RD	4.14	1.24, 13.85	†	
Four of five	1.96	1.04, 3.69	3.11	1.73, 5.60
Three of five	2.03	1.38, 2.98	1.35	0.76, 2.40
Two of five	1.62	1.22, 2.14	1.33	0.96, 1.85
One of five	1.55	1.22, 1.96	1.22	0.95, 1.57
HT-MSDs-DM-MD-RD	2.77	0.54, 14.33	†	
Four of five	1.86	1.07, 3.23	1.27	0.28, 5.68
Three of five	1.68	1.22, 2.31	2.38	1.56, 3.65
Two of five	1.84	1.41, 2.40	1.40	1.01, 1.93
One of five	1.42	1.13, 1.79	1.30	1.00, 1.68

^*∗*^Multivariate Cox proportional hazard models adjusted by age, education, marital status, living arrangement, self-rated health, visual impairment, and mini-mental score. ^a^Referent: no disease. HT: hypertension; MSDS: musculoskeletal disorders; CD: cardiovascular diseases; DM: diabetes mellitus; MD: mental disorders; RD: respiratory diseases. † denotes no value.

## Data Availability

All data generated or analysed during this study are included in this published article.
